# Supplementary material for: Network anomaly detection using Deep Autoencoder and parallel Artificial Bee Colony algorithm-trained neural network
Source: PeerJ Comput Sci. 2024 Oct 8;10:e2333. doi: 10.7717/peerj-cs.2333 (PMC13046298; doi:10.7717/peerj-cs.2333)
Supplement: Supplemental Information 2 [file peerj-cs-10-2333-s002.docx]

Dear Editorial Board Members and Peer Reviewers,

We are writing to provide detailed, valid reasons for the addition of Dr. Burcu Bakır-Güngör as an author to our manuscript titled "Network Anomaly Detection using Deep Autoencoder and Parallel Artificial Bee Colony Algorithm-Trained Neural Network". This letter serves to address the contributions made by Dr. Bakır-Güngör during the revision process and to justify her inclusion as a co-author.

**Justification for Addition of Dr. Burcu Bakır-Güngör**

**Contribution Timeline:** Dr. Burcu Bakır-Güngör became actively involved in the project following the initial submission of our manuscript, specifically during the revision phase that commenced after receiving the revision decision on February 8, 2024.

**Contributions:** Dr. Bakır-Güngör's contributions to the revision have been substantial and are detailed as follows:

1. **Experimental Design and Execution:**
   - Dr. Bakır-Güngör conducted new experiments that were essential for addressing the reviewers' comments regarding the robustness of our findings. Her expertise in machine learning was critical in designing and implementing these experiments.
2. **Data Analysis:**
   - She performed comprehensive data analysis on the newly acquired experimental results. Her analytical skills significantly enhanced the interpretation of our data, leading to more robust and reliable conclusions.
3. **Manuscript Writing and Editing:**
   - Dr. Bakır-Güngör made substantial contributions to the writing and editing of the revised manuscript. She revised several sections, including all sections, ensuring clarity, coherence, and scientific accuracy.
4. **Response to Reviewers:**
   - She actively participated in formulating detailed responses to the reviewers' comments. Her input was pivotal in addressing specific technical and theoretical issues raised by the reviewers.

**Reason for Omission in Initial Submission:** Dr. Bakır-Güngör was not involved in the initial submission due to her engagement in other professional commitments at that time. However, upon the receipt of the reviewers' feedback and recognizing the need for additional expertise, we invited her to join our team for the revision process. Her prompt and invaluable contributions during this phase warranted her inclusion as an author.

**Tracking Contributions in Revised Manuscript**

In the revised manuscript, we have used the commenting functionality to indicate which parts of the new text resulted from the involvement of Dr. Bakır-Güngör. These annotations clearly show her contributions to the experimental design, data analysis, and manuscript revisions.

We believe that the inclusion of Dr. Bakır-Güngör as a co-author aligns with the authorship criteria set forth by PeerJ and reflects the collaborative nature of our research efforts. We kindly request the Editorial Board and reviewers to consider this addition favorably.

Thank you for your attention to this matter. We remain at your disposal for any further information or clarification required.

Sincerely,

Hilal HACILAR
Abdullah Gul University
